# Anti-allodynic effect of intrathecal processed Aconitum jaluense is associated with the inhibition of microglial activation and P2X7 receptor expression in spinal cord

**DOI:** 10.1186/s12906-016-1201-2

**Published:** 2016-07-13

**Authors:** Jihoon Yang, Keun Suk Park, Jae Joon Yoon, Hong-Beom Bae, Myung Ha Yoon, Jeong Il Choi

**Affiliations:** Department of Biomedical Sciences, Chonnam National University Medical School, Gwangju, South Korea; Department of Anesthesiology and Pain Medicine, Chonnam National University Medical School and Hospital, Gwangju, South Korea; Center for Creative Biomedical Scientists at Chonnam National University, Gwangju, South Korea

**Keywords:** Allodynia, Aconitum jaluense, Microglia, P2X7 receptor

## Abstract

**Background:**

For their analgesic and anti-arthritic effects, Aconitum species have been used in folk medicine in some East Asian countries. Although their analgesic effect is attributed to its action on voltage-dependent sodium channels, they also suppress purinergic receptor expression in dorsal root ganglion neurons in rats with neuropathic pain. In vitro study also demonstrated that the Aconitum suppresses ATP-induced P2X7 receptor (P2X7R)-mediated inflammatory responses in microglial cell lines. Herein, we examined the effect of intrathecal administration of thermally processed Aconitum jaluense (PA) on pain behavior, P2X7R expression and microglial activation in a rat spinal nerve ligation (SNL) model.

**Methods:**

Mechanical allodynia induced by L5 SNL in Sprague-Dawley rats was measured using the von Frey test to evaluate the effect of intrathecal injection of PA. Changes in the expression of P2X7R in the spinal cord were examined using RT-PCR and Western blot analysis. In addition, the effect of intrathecal PA on microglial activation was evaluated by immunofluorescence.

**Results:**

Intrathecal PA attenuated mechanical allodynia in a dose-dependent manner showing both acute and chronic effects with 65 % of the maximal possible effect. The expression and production of spinal P2X7R was increased five days after SNL, but daily intrathecal PA injection significantly inhibited the increase to the level of naïve animals. Immunofluorescence of the spinal cord revealed a significant increase in P2X7R expression and activation of microglia in the dorsal horn, which was inhibited by intrathecal PA treatment. P2X7R co-localized with microglia marker, but not neurons.

**Conclusions:**

Intrathecal PA exerts anti-allodynic effects in neuropathic pain, possibly by suppressing P2X7R production and expression as well as reducing microglial activation in the spinal cord.

## Background

The *Aconitum* plants have shown analgesic effects in animal models of inflammatory and neuropathic pain.[1, 2] They also have been used in folk medicine for analgesic and anti-rheumatic effect, and for neurologic indications in Eastern Asia.[3] Aconitum, used without any processing, can cause serious toxicities such as cardiotoxicity and nephrotoxicity. Therefore, aconitum plants are generally detoxified through thermal processing before their use. Despite the traditional use of this plant for analgesia, the detailed mechanism attributable to its effect remains unclear.

Blockade of voltage-dependent sodium channels of neurons is considered the primary mechanism underlying the analgesic effects Aconitum plants.[4–6] However, spinal glial cells also play a significant role in the development and maintenance of neuropathic pain in addition to the involvement of neurons.[7, 8] Interestingly, lappaconitine, one of the alkaloid components of Aconitum plant species, was shown to suppress the expression of purinergic receptor in dorsal root ganglion neurons of rats with neuropathic pain.[9] If the processed Aconitum plant inhibits the expression of purinergic receptor on microglia, it is possible that it may inhibit microglial activation, thereby contributing to the attenuation of neuropathic pain. Supporting this hypothesis, a previous in vitro study demonstrated that Bullatine A, a diterpenoid alkaloid of the genus Aconitum, suppressed adenosine triphosphast (ATP)-induced P2X7R-mediated inflammatory responses in BV-2 microglial cells.[10] Indeed, activation of the P2X7R expressed on resting microglia is important for microglial activation in neuropathic pain [11–13].

This study was designed to examine the effect of intrathecally administered processed Aconitum jaluense (PA) on pain behavior and changes in the expression of P2X7R and microglial activation in an L5 spinal nerve ligation model (SNL) in rat.

## Methods

All experiments were performed in accordance with the International Association for the Study of Pain guidelines for the Use of Animals in Research. The protocol (CNU IACUC-H-2013-19) was approved by the Institutional Animal Care and Use Committee.

### Animal preparation

Male Sprague–Dawley rats weighing 225–250 g were housed in a room maintained at a constant temperature of 22–23 °C with an alternating 12 h light/dark cycle. Access to both water and food was provided *ad libitum*.

### Intrathecal catheter implantation and neuropathic pain model

A polyethylene-5 (PE-5) catheter was implanted into the intrathecal (i.t.) space for experimental drug administration as described previously.[14] Under general anesthesia using sevoflurane, a PE-5 catheter was introduced through the atlanto-occipital membrane and advanced caudally 8.5 cm to the level of the lumbar enlargement. Any rat with a neurological deficit after catheter implantation was killed immediately with an overdose of inhalation anesthetic. Animals were housed in individual cages following surgery.

After a recovery period of five days, L5 SNL was performed as described previously.[15] Briefly, the left L5 spinal nerve was isolated adjacent to the vertebral column during sevoflurane anesthesia and tightly ligated with a 6-0 silk suture distal to the dorsal root ganglia.

### Preparation and administration of experimental agents

Processed Aconitum jaluense (PA) was obtained from the Plant Extract Bank (PEB) at the Korea Research Institute of Bioscience and Biotechnology. Aconitum jaluense is one of the Aconitum species found in remote mountainous regions of the Korean peninsula, and it has been used for Korean folk medicine.[16, 17] The root of Aconitum jaluense was boiled in water at 100 °C for 150 min, followed by filtering and yield testing. After the extract was concentrated and dried at 75 °C, it was stored in a vacuumed bottle prior to use at -4 °C. The extract was dissolved with saline immediately before administration. I.t. administration was performed using a hand-driven, gear-operated syringe pump. All drugs were delivered in a volume of 10 μL, followed by an additional 10 μL of normal saline to flush the catheter. I.t. injection of PA was performed daily beginning from day 3 through day 7 following SNL.

### Behavioral study

By applying calibrated von Frey filaments (Stoelting, Wood Dale, IL, USA) having buckling forces between 0.41 and 15.2 g to the hind paw of animals, the paw withdrawal threshold (PWT) with 50 % probability was obtained using the up and down method, as described previously.[18] Mechanical allodynia was defined as having a PWT less than 4 g. Animals that did not show mechanical allodynia following SNL were excluded from this study.

On the day of the experiment to determine the acute effects of i.t. PA, rats were randomly allocated into experimental (PA; 10, 30, 100, and 300 μg, *N* = 8 per group) and control (saline, *N* = 8) groups. PWT was measured for 3 h at 15, 30, 60, 90, 120, 150, and 180 min following a single i.t. administration of PA or saline. The chronic effect of i.t. PA (300 μg, *N* = 8 per group) was also evaluated in another set of animals, who were injected daily for five days beginning from day 3 to day 7 after SNL; the PWT was measured 10 min before and 30 min after each injection. All experiments were performed by investigators blinded to treatment conditions.

### Western blot analysis

The spinal cord was rapidly removed by hydro-extrusion under inhalational anesthesia with sevoflurane (*N* = 7 per group). Lumbar enlargement of L4 –L6 was obtained, immediately frozen and stored at –70 °C. Collected tissue samples were homogenized in lysis buffer containing a mixture of protease inhibitors. Protein concentrations were determined, and aliquots (10 μg) were loaded and run on 12 % sodium dodecyl sulfate polyacrylamide gel electrophoresis (SDS–PAGE) gel, and electrophoretically transferred onto polyvinylidene difluoride (PVDF) membranes. Blots were blocked with 5 % non-fat milk in Tris-buffered saline (pH 7.5) containing 0.1 % Tween 20 for 1 h, and incubated overnight at 4 °C with rabbit anti-P2X7R (1:1000; Millipore).

After washing, blots were incubated with horseradish peroxidase-conjugated donkey anti-rabbit IgG (1:1000; Cell Signaling, USA) for 2 h at 4 °C, developed in enhanced chemiluminescent solution (Millipore) for approximately 1 min, and exposed onto film. Densities of specific P2X7R bands were using a computer-assisted imaging analysis system and were normalized against corresponding β-actin (1:1000; Cell Signaling) levels as a sample loading control.

### Determination of P2X7R expression by RT-PCR

The expression of P2X7R mRNA in the spinal cord was determined using reverse transcriptase polymerase chain reaction (RT-PCR, *N* = 7 per group). On days 3, 5 and 7 after SNL, ipsilateral dorsal spinal cord was obtained from the lumbar spinal enlargement, and total RNA was extracted with RNAiso Plus (Takara Bio, Japan). RNA yield was determined by measuring the absorbance at 260 and 280 nm. RT-PCR was performed using a PrimeScript RT-PCR Kit (Takara Bio) according to the manufacturer’s protocol.

Total RNA (1 μg) was reverse-transcribed for 60 min at 42 °C, and PCR amplification was performed for the P2X7R for 34 cycles of denaturation (95 °C for 1 min), annealing (65 °C for 30 s) and extension (72 °C for 1 min). For GAPDH, amplification was performed in 23 cycles of denaturation (95 °C for 30 s), annealing (55 °C for 30 s) and extension (72 °C for 30 s). PCR products were separated on 1.5 % agarose gels and made visible using SYBR safe DNA gel stain. Band intensity was measured by densitometry, and relative values of the P2X7R to GAPDH were calculated. Primers coding for rat P2X7R and GAPDH were designed as follows: P2X7R, forward 5’-AAT GAG TCC CTG TTC CCT GGC TAC-3’ and reverse 5’-CAG TTC CAA GAA GTC CGT CTG G-3’; GAPDH, forward 5’-GGC CTT CCG TGT TCC TAC C-3’ and reverse 5’-CGG CAT GTC AGA TCC ACA AC-3’.

### Immunofluorescence

Rats were deeply anesthetized by intraperitoneal injection of a mixture of ketamine and xylazine (4:1, 0.7 ml/kg), and perfused transcardially with 200 mL of 0.01 M phosphate-buffered saline (PBS, pH 7.4), followed by 300 mL of 4 % paraformaldehyde in 0.1 M phosphate buffer (pH 7.4). After the perfusion, lumbar enlargement segments were harvested (*N* = 4 per group) and post-fixed in the same perfusate at 4 °C for 6 h. Tissues were immersed in 20 % sucrose solution for 12 h and then in 30 % sucrose solution overnight until they sank to the bottom for cytoprotection. After snap-freezing, transverse sections (20 μm) were obtained using a cryostat.

All sections were blocked with 3 % normal goat serum in 0.3 % Triton X-100 for 2.5 h at room temperature. Sections were subsequently incubated with rabbit polyclonal antibody against P2X7R (1:200, Millipore) or mouse monoclonal antibody Iba-1 (microglial marker, 1:200; Abcam, UK) for 4 °C overnight.

Antibody binding to tissue sections was visualized with a mixture of goat anti-rabbit IgG conjugated with Alexa Fluor 488 (1:1000; Invitrogen, USA) or goat anti-mouse IgG conjugated with Alexa Fluor 594 (1:1000; Invitrogen) for 2 h at 37 °C. Sections were rinsed in 0.01 M PBS and cover slips were applied.

For double-label immunofluorescence, spinal cord sections were incubated with 3 % normal goat serum for 30 min at room temperature. Sections were subsequently incubated with rabbit polyclonal antibody against P2X7R (1:200; Millipore) at 37 °C for 1 h, and then 4 °C overnight. Antibody binding to tissue sections was visualized with FITC labeled anti-rabbit IgG conjugated with Alexa Fluor 488 for 1.5 h at 37 °C. Sections were then incubated with 3 % normal goat serum and mouse monoclonal anti- Iba-1 (1:200; Abcam) or mouse monoclonal anti-NeuN Ab (1:1000; Millipore) for 1 h at 37 °C. Bilateral images were captured with a fluorescence microscope at 10–60X. Acquired images were imported into Adobe Photoshop software for image presentation. Images were then converted to gray scale and analyzed using Image J software. After drawing an outline on the dorsal horn gray matter, fluorescent intensity was obtained using the same outline for each image and was subtracted by background intensity.

### Statistical analysis

Data are expressed as the means ± standard error of the mean (S.E.M). Dose-response data are presented as a percentage of the maximum possible effect, calculated as follows: (post-drug PWT – post-injury baseline PWT)/(cut-off PWT – post-injury baseline PWT)] × 100. We used one-way analysis of variance (ANOVA) with Bonferroni correction for data analysis, but performed Krulskal-Walli's test followed by Dunn's post hoc test for immunofluorescence and unpaired *t*-test for data on the chronic analgesic effect of i.t. PA. Results were considered statistically significant when *P* < 0.05.

## Results

**Fig. 1:**
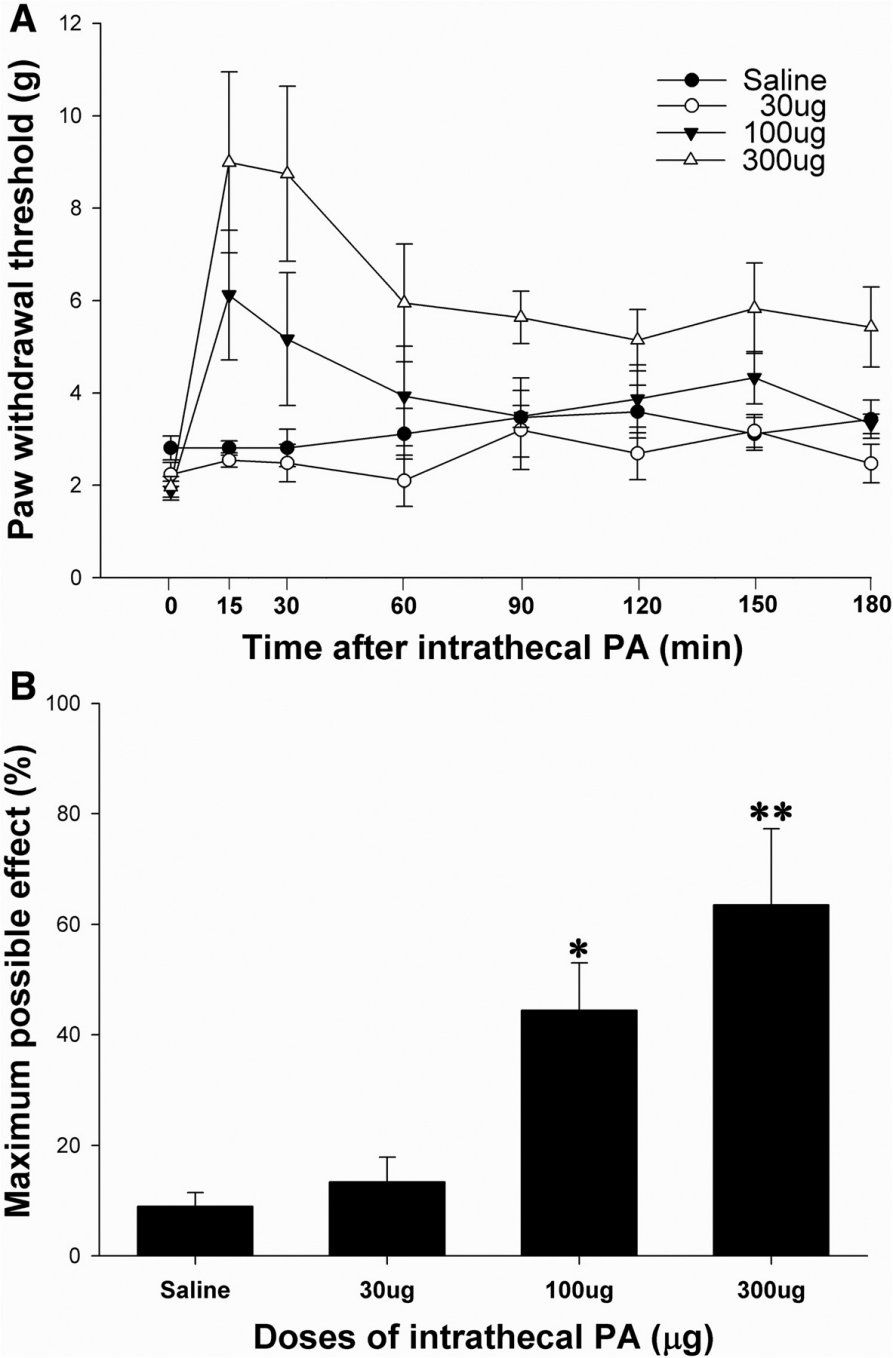
Acute effects of intrathecal (i.t.) processed Aconitum jaluense (PA) on the hind paw withdrawal response to von Frey filaments following spinal nerve ligation (SNL). Time course of paw withdrawal threshold (**a**) shows an increase in PWT in animals treated with i.t. PA, which peaks within 30 min. The anti-allodynic effect lasted 3 h following i.t. PA. The percentage of maximal possible effect (**b**) of i.t. PA was 65 % at a dose of 300 μg. *N* = 8 per each treatment. *P < 0.05, **P < 0.01 vs. saline

**Fig. 2:**
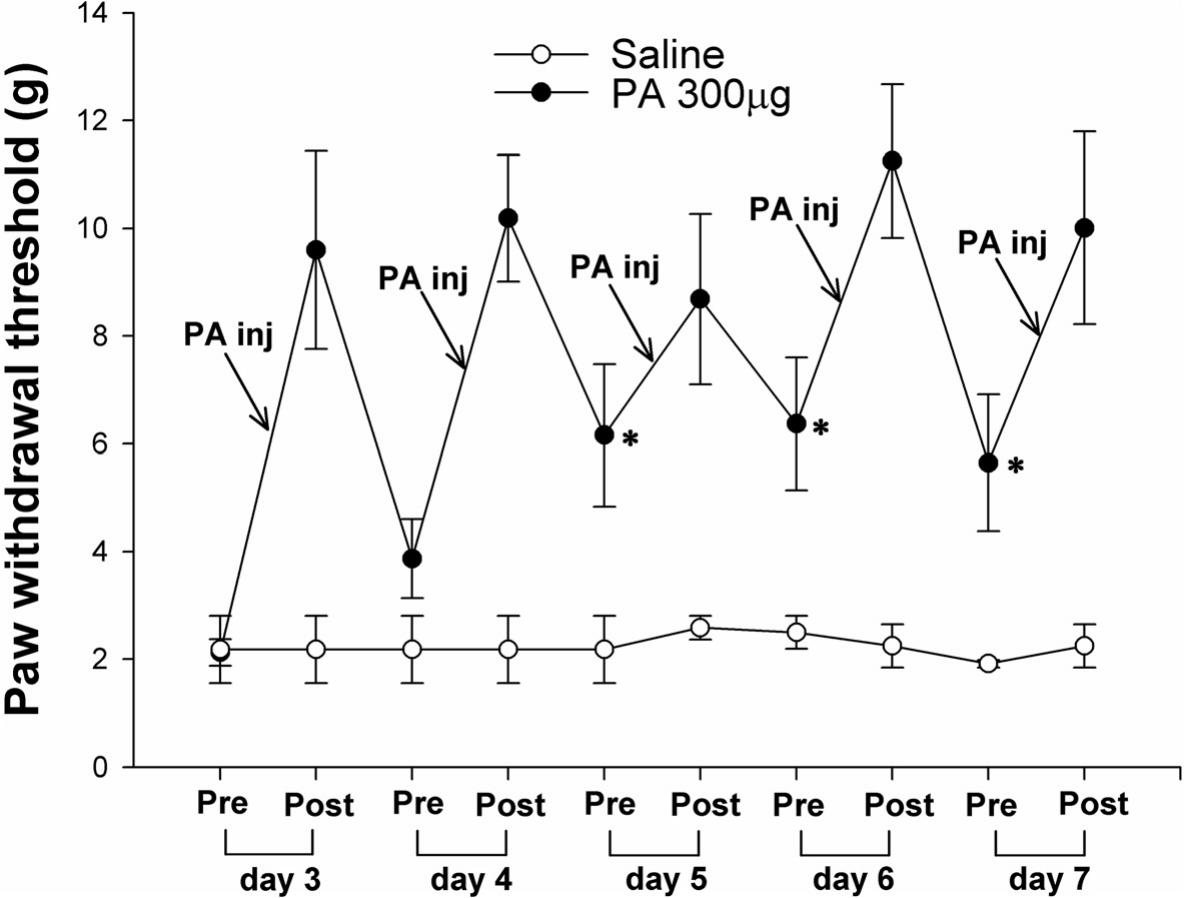
Chronic effects of intrathecal (i.t.) PA on allodynic response to von Frey filaments following spinal nerve ligation (SNL). Each group received i.t. PA (*N* = 8) or saline (*N* = 8) once daily for 5 days. ‘Pre’ and ‘Post’ on the x-axis represent the PWT measured 10 min before and 30 min following i.t. PA injection, respectively. *P < 0.05 vs. corresponding PWT of the saline group measured prior to injection

Acute single i.t. PA treatment produced a significant anti-allodynic effect in SNL animals in a dose-dependent manner compared with control (saline) group (Fig. 1). Its effect peaked within 30 min (MPE 65 %), then decreased, and lasted until 3 h after i.t. treatment. Daily i.t. PA for five days attenuated the mechanical allodynia compared to the control group (Fig. 2). Interestingly, the PWT measured before i.t. PA on days 5 and 7 was also significantly higher than the corresponding control group, indicative of a chronic anti-allodynic effect of i.t. PA.

**Fig. 3:**
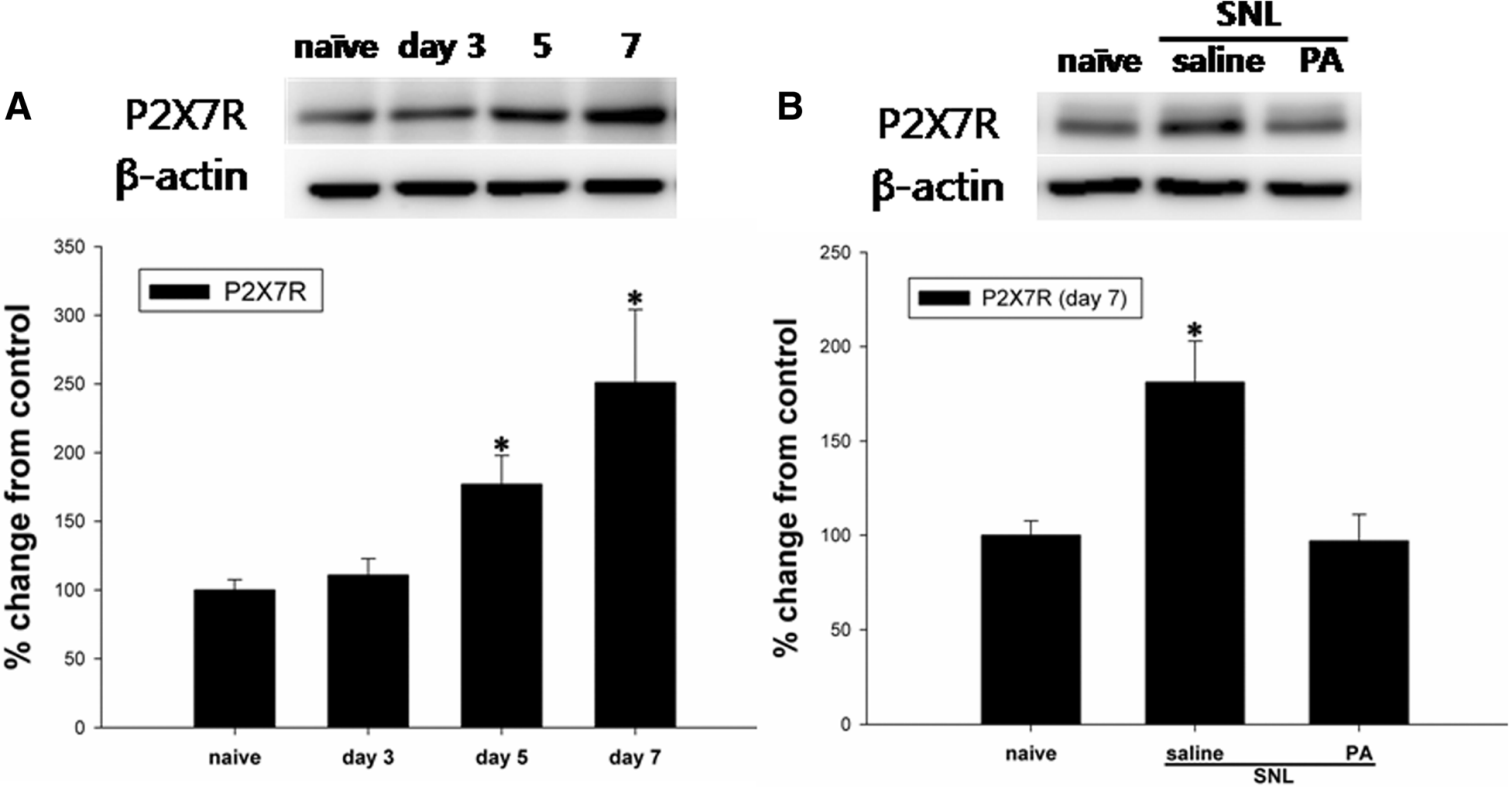
**a** Changes in P2X7 receptor (P2X7R) expression in the spinal cord ipsilateral to the SNL by Western blot analysis. Bar graphs depicting the relative intensity of P2X7R expression show a gradual increase in P2X7R levels in the ipsilateral dorsal spinal cord. **b** Effect of intrathecal (i.t.) administration of PA on P2X7R expression in the ipsilateral dorsal spinal cord. Representative blots showing the expression of P2X7R and GAPDH on day 7 following SNL. Intrathecal injection of PA or saline was performed from days 3 to 7. The increase in P2X7R in the ipsilateral dorsal spinal cord was significantly blocked in animals treated with i.t. PA (300 μg). *N* = 7 per group. *P < 0.05 vs. naive

**Fig. 4:**
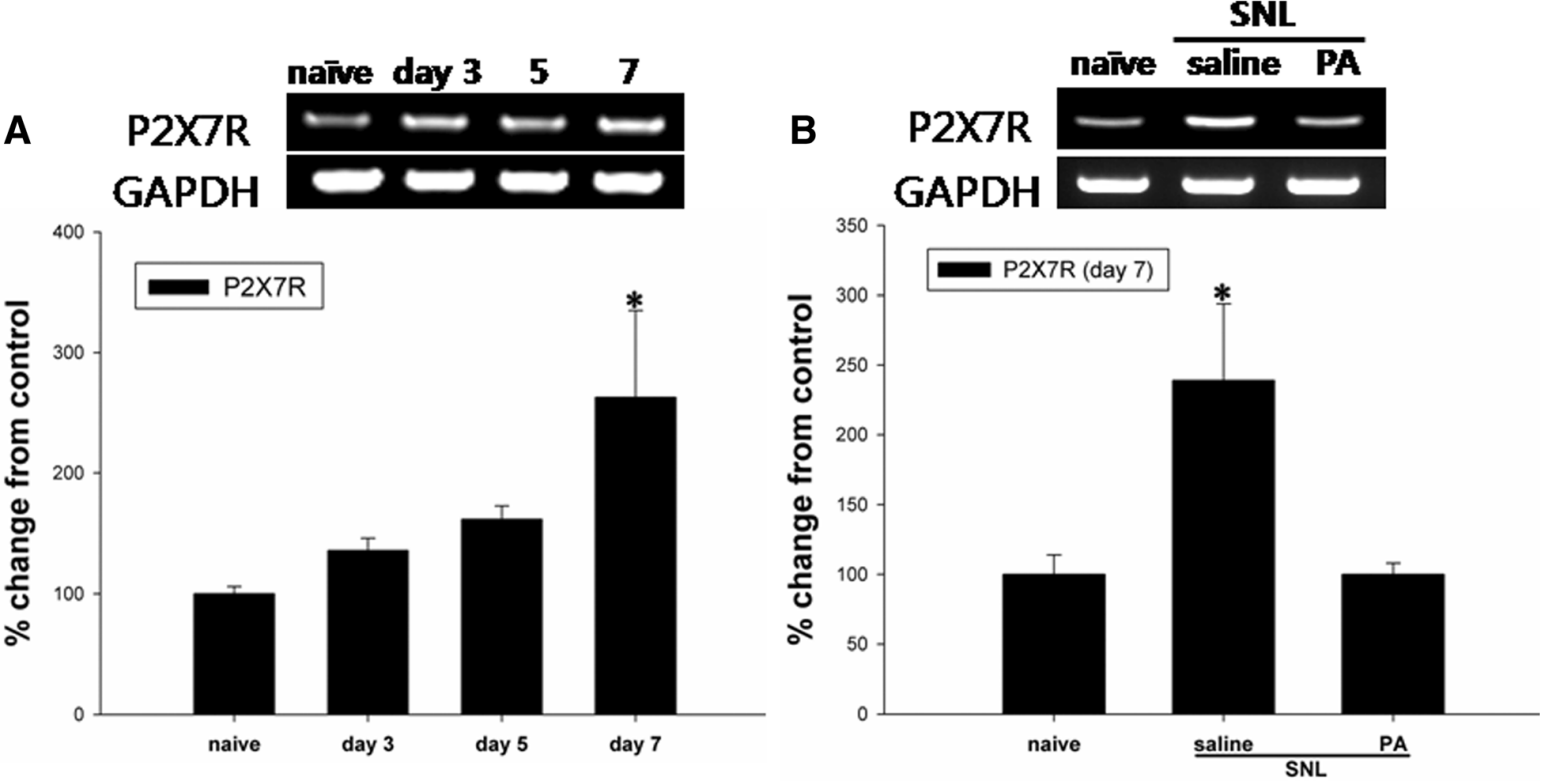
**a** Increased expression of P2X7R mRNA in the ipsilateral dorsal spinal cord following L5 SNL. Gel panels show RT-PCR products from the spinal cord of naïve rats on days 3, 5 and 7 following SNL. P2X7R mRNA is significantly increased after SNL compared to naïve animals. **b** Intrathecal (i.t.) administration of PA blocked the increase in P2X7R mRNA expression in the ipsilateral dorsal spinal cord on day 7 following SNL. *N* = 7 per group. *P < 0.05 vs. naive

Protein levels of spinal P2X7R gradually increased in SNL animals compared to naïve rats, and peaked seven days after SNL. Its increase was significantly attenuated in animals treated with daily i.t. PA (Fig. 3). In addition, mRNA expression of P2X7R was determined to significantly increase on day 7 after the injury, which was obliterated by i.t. PA treatment (Fig. 4).

**Fig. 5:**
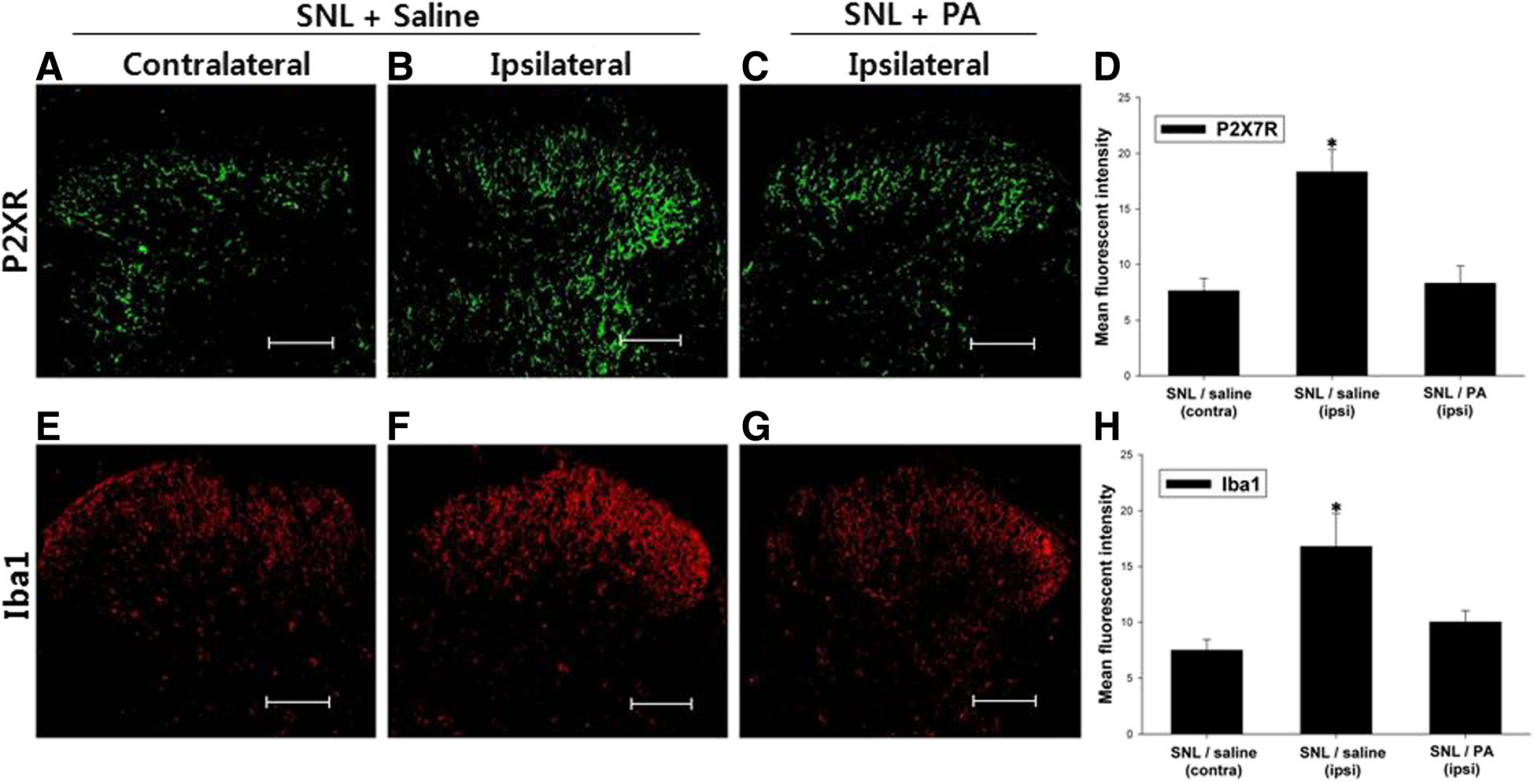
Intrathecal (i.t.) PA attenuated the increase in P2X7R and Iba-1 expression in the dorsal horn of the spinal cord seven days following L5 SNL. P2X7R immunoreactivity (green) was increased on the ipsilateral side (**b**) compared with the contralateral side (**a**). Expression of Iba1 in microglia (red) within the dorsal horn was significantly increased on the ipsilateral side (**f**) compared with the contralateral side (**e**). Both the increase in immunoreactivity to P2X7R and Iba-1 were attenuated by i.t. PA treatment (**c**, **g**), which was analyzed and depicted by bar graphs showing mean fluorescence intensity (**d**, **h**). *N* = 4 animals/group for all bars. Scale bar, 25 μm. *P < 0.05 vs. SNL/PA

**Fig. 6:**
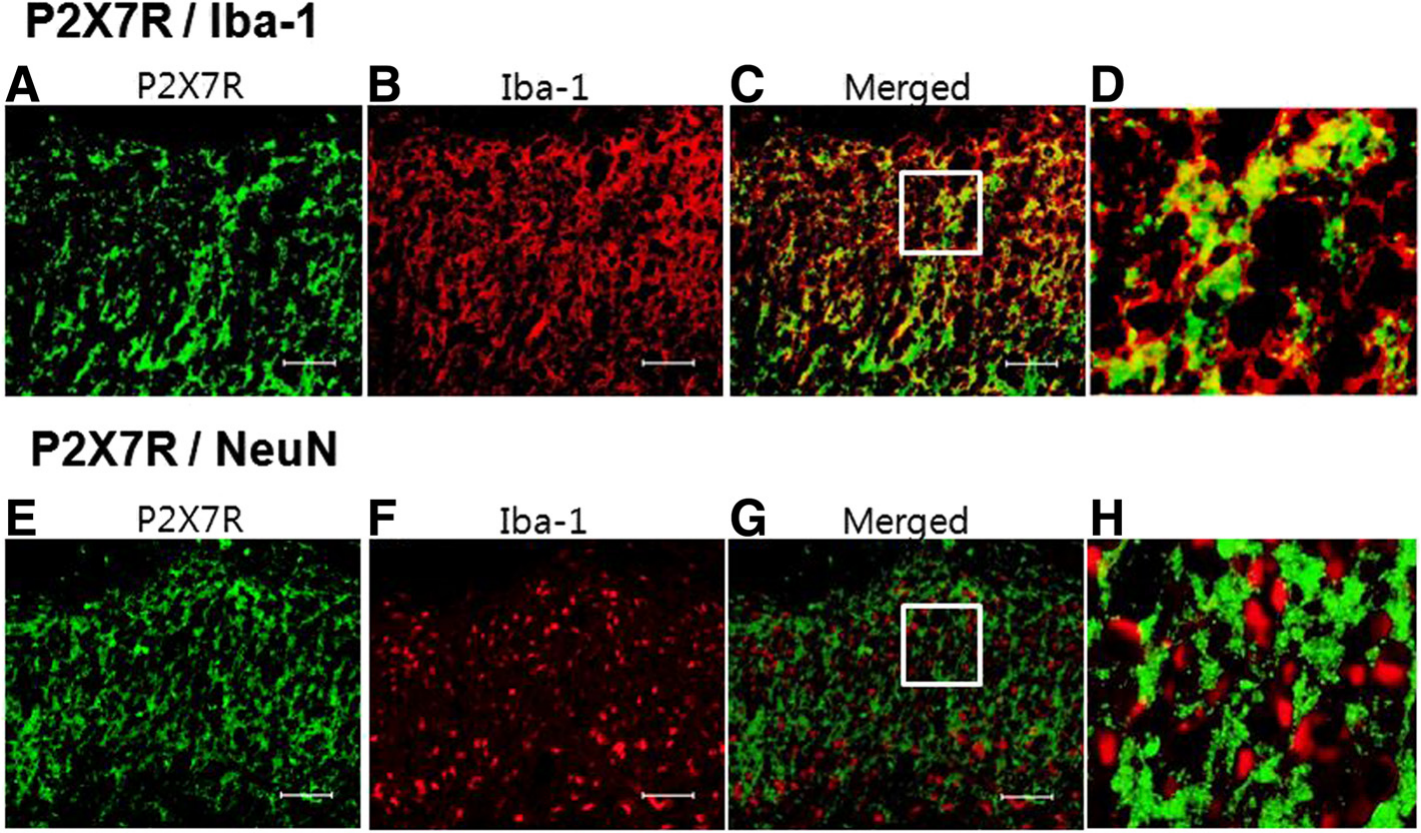
Representative sections double labeled for anti-P2X7R (**a**, **e**) with anti-Iba1 (**b**) or anti-Neu-N (**f**) of the spinal dorsal horn on day 7 following L5 SNL. Right photographs (**d**, **h**) show amplified images derived from white open squares in merged images (**c**, **g**), indicating that P2X7R is expressed exclusively in microglia. Scale bar, 25 μm

Immunoreactivity to Iba1, a microglial marker, was significantly increased in the ipsilateral spinal dorsal horn on day 7 after SNL, indicative of microglial activation. In parallel, immunoreactivity to P2X7R of the ipsilateral dorsal horn was significantly increased compared to the contralateral dorsal horn. Daily i.t. PA significantly reduced changes in immunoreactivity to Iba-1, as well as P2X7R (Fig. 5). In addition, P2X7R co-localized with Iba-1, but not Neu-N, suggesting that expression of the increased mainly on microglia, rather than neurons in the spinal dorsal horn of SNL animals (Fig. 6).

## Discussion

This study demonstrated that the PA exerts anti-allodynic effects and suppresses microglial activation, as well as the expression of P2X7R on microglia in the spinal cord in neuropathic pain rat model.

Over 250 species make up the genus Aconitum plant, which belongs to the family Ranunculaceae. Previous studies have reported several beneficial effects of PA that are associated with its traditional use as an herbal medicine. It has been used for its analgesic, anti-inflammatory, and neurological effects [2, 4] However, the adverse effects and undefined mechanisms limited its use as a medicinal drug.[19, 20] Aconitum jaluense is one of the Aconitum plants found in the Korean peninsula, and has been used for analgesic effect and alleviating inflammatory symptoms of arthritis.[16, 17] In addition to its traditional use for reducing inflammation and producing analgesia for arthritis, it also attenuated the allodynic response in an animal neuropathic pain model in the present study.

Several mechanisms underlying these effects of Aconitum have been proposed; activation of spinal k-opioid receptor, inhibiting the growth of sympathetic sprouting in dorsal ganglia, and down regulation of purinergic receptors.[9, 21] However, the voltage dependent sodium channel is considered a primary target for the analgesic and side effects of Aconitum plants.[4–6, 19] Therefore, the anti-allodynic effect of i.t. PA shown in the current study could be attributed to its action on the sodium channel on neurons in the spinal cord. Previous studies demonstrated that the nociceptive behaviors in acute and inflammatory pain models, including hot plate test and carrageenan model, decreased in animals treated with processed Aconitum plant. [6, 22] In the current study, i.t. PA produced not only acute, but also chronic anti-allodynic effects following daily i.t. treatment beginning on day 3 after SNL. The intensity of allodynia was reduced on days 5 and day 7 even before i.t PA administration. The mechanism of the chronic effect remains unclear, but its action on the sodium channel on neuron may not be the sole mechanism responsible for the anti-allodynic effect. As shown in the current study, daily i.t. PA significantly blocked both increase of P2X7R expression and microglial activation. This finding suggests that the anti-allodynic, in particular chronic effect is associated with the effect of i.t. PA on microglia and P2X7R.

Meanwhile, it is known that glia, as well as neurons in the central nervous system, play a critical role in the development and maintenance of neuropathic pain.[7, 8] Many studies have suggested that microglia in the spinal cord are activated in response to peripheral nerve injury participating in the pathogenic mechanisms of neuropathic pain.[23, 24] The current study demonstrated that the pretreatment with PA significantly attenuated the activation of microglia which occurs in response to peripheral nerve injury induced by spinal nerve ligation. Inhibition of microglial activation could be caused by direct effect of PA blocking sodium channel or purinergic receptor on microglia. However, the attenuation of microglial activation might be caused indirectly, possibly by blocking the interaction between neurons and microglia.[7, 8] The sodium channel blocking effect could have resulted in less intense firing of neurons involved in peripheral nerve injury, and subsequent inhibition of activation of microglia.[25, 26] The causal relationship between anti-allodynic effect of i.t. treatment of PA and microglial activation should be further explored to elucidate the mechanism underlying its anti-allodynic effect.

Purinergic signaling is one of the key players in microglial activation and its interaction with neurons.[27, 28] Limited information has been provided regarding the role of the purinergic receptor in the analgesic effect produced by Aconitum plants. P2X3 receptors are known to be involved in producing analgesic effect of lapaconitine, a type of aconitum alkaloid.[9] Lapaconitine downregulated the P2X3 receptor expression in dorsal root ganglion neurons in a chronic constriction injury rat model, suggesting that some of principles of Aconitum have a substantial effect on puringergic receptors, in particular, P2X ionotropic receptors.

The expression of P2X receptors on microglia is mainly confined to P2X4 and P2X7Rs.[11] In the present study, the expression of P2X7R was observed in microglia, but not in neurons. A previous in vitro study also investigated the effect of the genus Aconitum on the function of microglia using the BV-2 microglia cell line, in which Bullatine A, an alkaloid of Aconitum, inhibited ATP-induced BV-2 cell death and apoptosis and inflammatory responses by suppressing P2X7R expression.[10] In the current study, overall expression of the P2X7R in the ipsilateral spinal dorsal horn was significantly attenuated by i.t. PA, compared to control animals in which P2X7R expression increased gradually following the ligation of the spinal nerve. Previous experiments also have shown that P2X7R is expressed mostly in microglia which was activated in response to injury.[12, 27, 29] These findings suggest a direct effect of i.t. PA on microglia resulting in suppression of P2X7R expression. The result of current study warrant further investigations on the active analgesic ingredients of Aconitum jaluense and its possible pharmacologic actions on purinergic receptors, including P2X receptors.

## Conclusions

Intrathecal (i.t.) PA exerts both acute and chronic antiallodynic effect in neuropathic pain induced by SNL. Microglia activation and expression of P2X7R in the spinal cord are inhibited by i.t. PA, and these effects may contribute to antiallodynic effect of i.t. PA.
